# Supplementary material for: The Highly Leukotoxic JP2 Genotype of Aggregatibacter actinomycetemcomitans Is Present in the Population of the West African Island, Sal in Cape Verde: A Pilot Study
Source: Pathogens. 2022 May 13;11(5):577. doi: 10.3390/pathogens11050577 (PMC9145930; doi:10.3390/pathogens11050577)
Supplement: Supplementary file 1 [file pathogens-11-00577-s001.zip › pathogens-1694892-supplementary.pdf]

Table S1. Clinical and microbiological data from all participants.

Sample #	Relationship	Gender	Age	Oral pain	Brushing frequency	Smoking cigarettes	Teeth present	DMFT	CPI highest	Log Aa/mL in plaque	Log Aa/mL in saliva
2	unrelated	M	48	Yes	3	yes	15	4	4	4.38	
3	partner of 4	M	54	No	2	yes	10	4	3	4.48	2.76
4	partner of 3	F	50	Yes	2	yes	19	2	4	4.46	
5	partner of 9	M	51			yes	18	5	4	5.2	
6	grandfather of 7	M	57	No	3	no	25	0	4	4.51	
8	unrelated	F	53	Yes	3	no	17	1	4	4.64	3.38
9	partner of 5	F	54		3	no	20	2	4	6.04	
10	unrelated	M	31	Yes	3	no	28	4	2	4.28	
11	unrelated	M	37	No	2	no	23	0	3	4.23	2.08
12	unrelated	F	25	Yes	2	no	31	7	4	4.96	
13	unrelated	F	26	Yes	1	no	31	0	3	5.98	
14	unrelated	F	22	Yes	2	no	30	8	4	4.11	
15	unrelated	F	25	No	2	no	29	0	4	5.76	
16	unrelated	F	43	No	3	no	30	7	4	4.66	
17	unrelated	F	22	No	3	no	31	8	2	4.83	
18	unrelated	F	38	No	2	no	29	6	3	4.30	
19	unrelated	M	59	No	2	no	22	1	4	4.23	2.79
20	unrelated	M	40		3		31	1	4	5.69	
22	parent of 21	M	37			no	21	5	4	5.85	
23	brother 22	M	29			yes	18	10	3	4.18	
26	family of 22&23	F		Yes			23	10	2	3.60	
28	friend	M					19	3	4	4.38	

29	not related/ lives same appartement as 28	F	20	Yes	no	30	7	3	4.56	3.00 *
30	mother 22	F	56	No	no	8	0	4	5.20	3.48
34	friend of family; other island	M	40	No		28	3	3	4.11	
35	unrelated	F	39			14	3	3	4.48 *	3.28
38	mother 37	F	49	Yes	no	19	4	4	3.81	3.77
39	friend	F	25	No	yes	28	5	3	4.11	2.72
40	daughter 38	F	28			24	3	4	3.95	
median:							4.0	4	4.7	3.0
mean:			39			23			4.5	3.2

Figure S1: Data of the study population, with median or mean per item.
Demographical data contain, beside age and gender, also data on family relationships as far as could be obtained.
Brushing frequency and smoking are self-reported data and may be subjected to bias.
CPI is recorded with 0 as minimum and 4 as maximum.
DMFT is recorded on the basis of the number of teeth present, since tooth loss due to trauma, periodontitis or caries could not be differentiated.
Aa is the concentration *A. actinomycetemcomitans* in plaque or saliva as given in Log CFU/sample or Log CFU/mL respectively
*Means that these concentrations are for the JP2 genotype.

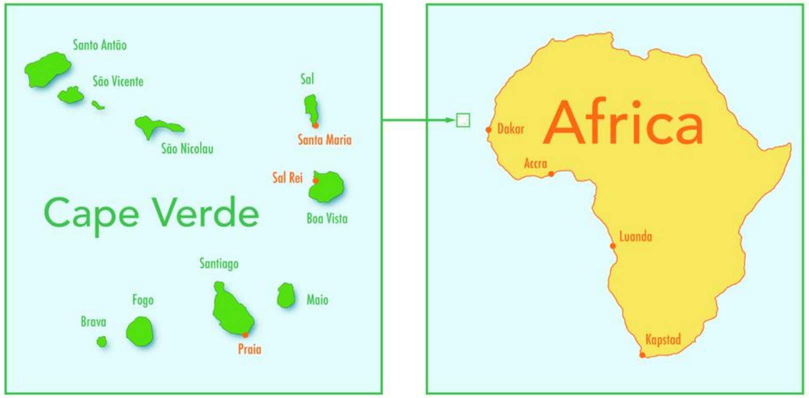

(a) (b)

Figure S1. The location of (**a**) Sal and (**b**) Cape Verde off the west coast of Africa.
